# Immersing healthcare students in a virtual reality Parkinson's disease experience

**DOI:** 10.5116/ijme.65f5.725c

**Published:** 2024-03-29

**Authors:** Yi (Joy) Li, Jason Orlosky, Wanda Jirau-Rosaly, Shilpa Brown, Nicole Rockich-Winston

**Affiliations:** 1 Kennesaw State University, Department of Software Engineering & Game Development, Marietta, Georgia, USA; 2 Augusta University, Department of Computer & Cyber Sciences, Augusta, Georgia, USA, and Osaka University, Cybermedia Center, Toyonaka, Japan; 3 Medical College of Georgia at Augusta University, Department of Medicine, Augusta, Georgia, USA; 4 Medical College of Georgia at Augusta University, Department of Pharmacology and Toxicology, Augusta, Georgia, USA

**Keywords:** Virtual reality, Parkinson’s disease, empathy, interprofessional education, simulation

## To the Editor

The aging population in the United States poses considerable challenges to our healthcare system.^1^ Co-morbidities such as movement disorders further complicate geriatric care. In particular, Parkinson’s disease is a neurodegenerative disorder characterized by slow movements, resting tremors, and/or rigidity due to the loss of dopaminergic neurons.^2^ Recent estimates indicate that at least 1% of the population of over 60 years old have been diagnosed with Parkinson’s disease.^2^ From a health systems perspective, patients with Parkinson’s disease require interprofessional, patient-centered care, including collaborations among physical therapists, occupational therapists, and physicians.^3^^-^^4^

Developing an appreciation for how activities of daily living (ADLs) can be affected by Parkinson’s disease is important to develop empathy and may also lead to better patient care and outcomes.^5^ Virtual reality (VR) offers an educational platform to engage learners in a variety experiences, including situations that could not be experienced otherwise like patients’ viewpoints.^6^^-^^8^ Several studies have described positive outcomes like improvement in participants’ perspectives and empathy development using VR experiences^6^^-^^8^ and illustrated the use of VR for the purposes of rehabilitation in patients with Parkinson’s disease.^9^ However, the development and analysis of an immersive VR Parkinson’s disease experience has yet to be described in an interprofessional context. As such, we created an interprofessional session for students to experience the challenges of living with Parkinson’s disease through VR and examined students’ perceptions of the experience focusing on the use of VR.

Pre-clerkship medical students, as well as first-year occupational (OT) and second-year physical therapy (PT) students at Augusta University, participated in a geriatric workshop in Fall 2021 as part of the medical student’s required Patient Centered Learning (PCL) course. PCL is a required course that takes place in the pre-clerkship phase within the medical school. The purpose of the PCL course is to teach students basic patient care skills. The 4-hour, single-day workshop consisted of six stations composed of different evaluations for a patient with Parkinson’s disease, one of which was the VR station. The other stations involved the following specialties, focusing on the care of a patient with Parkinson’s disease: physical therapy, occupational therapy, pharmacotherapy, cognitive deficits, and home health evaluations; medical, OT, and PT students worked collaboratively to evaluate the patient at these stations. The researchers developed a VR station in collaboration with computer science students and faculty as well as clinicians who treat patients with Parkinson’s disease, and each student within the group had the opportunity to experience the perspective of an older adult with Parkinson’s disease in a VR home environment.

The VR program and interface were previously described.^10^ Participants had to complete a series of activities tied to daily living, including showering and grooming. The immersive experience included narrative and interactive features and was designed using the Unity game engine. Students used the HTC Vive Pro VR headset during the session. Updated features incorporated in this workshop included an advanced eating feature and hallucinations. The added task would prompt participants to pour milk and cereal into a bowl and then use a spoon to eat the cereal. The hand tremor often made the task difficult to accomplish because the falling trajectory of the objects would amplify any movement of the hand, causing cereal to miss the bowl, fall out of the spoon, and scatter over the counter and floor. Participants were provided an option to use a weighted spoon to stabilize the hand and finish the task. Additionally, a hallucination feature was added to mirror how patients with Parkinson’s disease experience hallucinations due to medication side effects. During the aforementioned tasks, there were random chances that some objects in view would appear or disappear.

A voluntary post-survey asked students about their perceptions of the activity and the use of VR in healthcare education with a 5-point Likert scale (e.g. strongly agree, agree, neutral, disagree, strongly disagree) and open-ended questions immediately following the activity and remained open for one week. Anonymous data were collected voluntarily as a part of routine course evaluation processes. Students were offered an opportunity to participate in focus groups to discuss the geriatric workshop collectively and provide feedback on the VR station. Descriptive statistics, one-way analysis of variance (ANOVA), and Glaser’s constant comparative method were used to analyze data.^11^

In total, 194 medical, 45 OT, and 35 PT students participated in the workshop. Of these, 104/274 (37.8% response rate) completed the post-survey (75 medical, 17 OT, 12 PT). Additionally, two focus groups, each with four participants, asked the following with respect to the VR experience: (1) how has the virtual reality experience changed your perspective on patient’s living with Parkinson’s disease and (2) how do you view the utility of VR simulations in medical education?  Most students (98%) felt the VR experience increased their appreciation for how Parkinson’s disease affects ADLs. Many students felt the VR experience would help them empathize with patients (81%), and students indicated VR was a useful tool in health science education (77%).  Similar to a previous iteration of this workshop, empathy among participants may have been less affected due to high baseline empathy towards patients.^12^ Among the professions, there were no significant differences between respondents (p=0.407). Analysis of the textual data indicated that participants appreciated the realism/realistic shakiness of the VR controllers and empathizing with patients living with Parkinson’s disease.

With respect to our focus group data, two major themes were identified: (1) better understanding the challenges patients living with Parkinson’s disease face while trying to remain independent and (2) the unlimited possibilities of VR simulation in augmenting medical education. As one student described regarding his increased empathy towards patients living with debilitating diseases, “when I have a chance to work with these patients [patients living with Parkinson’s disease], I will remember how difficult it was for me to make myself a bowl of cereal or how much trouble I had dialing [on a] phone” (Participant 6). Additionally, another student described, “going through each ADL, I got more and more frustrated, and by the end, all I thought about was how in the world patients dealt with these difficulties” (Participant 2). Concerning the utility of VR simulations in medical education, one student noted, “I knew VR was used in surgery training and other specialties but wasn’t aware of empathy training. Thinking of all the disease states we learn about, we could do so much!” (Participant 1)

Immersing medical, occupational therapy, and physical therapy students into a simulated VR environment positively influences their perceptions of patients with Parkinson’s disease. The application mirrors the everyday tasks that can be affected by the consequences of neurodegenerative disorders. Our approach also demonstrates the synergy between computer science and medical faculty, resulting in a de novo VR program that centers on ADLs. Additionally, our next iteration will include data collection on changes in empathy simultaneously within the VR experience, asking participants to rate changes after each ADL activity. This Parkinson's disease VR simulation is both translatable and shareable to other healthcare science programs. Currently, VR simulations have been used in medical education to practice technical psychomotor skills, augment learner engagement, develop empathy, and train emergency providers.^13^ Immersive VR simulations have the potential to transform our capacity to develop empathetic and highly skilled physicians, favoring the approach of “learning by doing,” rather than traditional methods like didactic lectures and demonstrations. Our approach shows that providing opportunities for students to experience what it is like to live with health issues can positively influence their perceptions and empathy towards patients.

### Conflicts of Interest

The authors declare that they have no conflict of interest.
